# Supplementary material for: HiLAB: A Hybrid Inverse‐Design Framework
Source: Small Methods. 2025 Sep 20;9(11):e00975. doi: 10.1002/smtd.202500975 (PMC12641366; doi:10.1002/smtd.202500975)
Supplement: Supplementary file 1 — Supporting Information [file SMTD-9-e00975-s001.pdf]

Supplementary Information for

HiLAB: A Hybrid Inverse-Design Framework

S1 Empirical Analysis of TO Termination and Framework Comparison

To provide a rigorous, data-driven basis for our choice to terminate the partial topological optimization (TO) runs at 35 iterations, we performed a controlled analysis. The results, shown in Figure S1, empirically justify this decision as a strategic trade-off between design quality and the computational cost required to generate the VAE training dataset. For this analysis, all TO runs were performed with a fixed random seed (42) and consistent hyper-parameters for the cone filter ($r_{min} = 1.0, r_{max} = 2.5, k = 15$) and progressive binarization ($\alpha_{min} = 1.0, \alpha_{max} = 8.0, s = 10$), as defined in Equations (2) and (3) of the main text.

The plot clearly shows that the figure of merit (FoM) improvement curve reaches a distinct knee in the 30-40 iteration range, after which the performance gains diminish significantly while the simulation time continues to increase linearly. This empirical evidence supports our choice of 35 iterations as an optimal point to capture stable, high-performance design features without incurring the significant cost of full convergence. We emphasize that this value is not a universal constant; the optimal truncation point is problem-dependent and may vary for different device objectives or physical constraints.

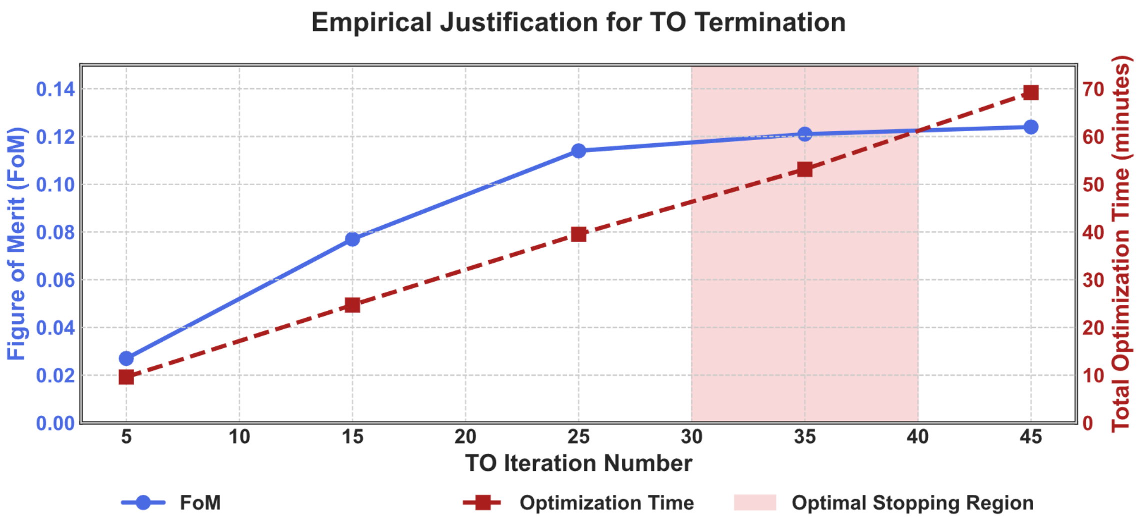

Figure S1: The evolution of the figure of merit (FoM, blue solid line, left axis) and the total computational time (red dashed line, right axis) as a function of the TO iteration number. The plot illustrates the trade-off between performance gain and computational cost. The FoM improvement curve begins to plateau around the 30–40 iteration mark (highlighted as the optimal stopping region), indicating diminishing returns for further simulations. This analysis confirms that terminating the TO at 35 iterations provides a computationally efficient point to capture stable, high-performance designs for the VAE training dataset.

To further provide a clear comparison between our method and a conventional approach, Table S1 provides a direct, quantitative summary. The data highlights the significant advantages of the HiLAB framework in terms of both computational efficiency and the quality of the final optimized design. As the table shows, the HiLAB framework not only reduces the required number of electromagnetic simulations by an order of magnitude but also finds superior solutions by avoiding the weak local optima that limit traditional TO. Furthermore, its ability to co-optimize both the geometric pattern and the physical device parameters in a single loop, coupled with the transferability of the trained VAE, makes it a more powerful and flexible platform for advanced nanophotonic design.

Table S1: **Comparison of HiLAB and Conventional Topology Optimization**

Metric	Conventional TO	HiLAB Framework	Advantage
**Total EM Simulations**	∼14,000 (70 restarts × 200 iterations)	∼1,400 (1,050 for VAE + 350 for BO)	>10× reduction
**Best Achieved FoM**	0.188 (from 70 TO runs, each with 200 iterations)	0.247	31.4% improvement
**Optimization Outcome**	Prone to weak local optima; sensitive to initial conditions	Systematic exploration of near-global optimum; robust	Superior solution quality
**Parameters Optimized**	Pattern only (physical parameters fixed)	Pattern and physical parameters (e.g., thickness, period)	Complete co-optimization
**Flexibility for New Tasks**	Requires complete re-runs from scratch	Reuses trained VAE; requires only a new BO run (∼350 sims)	Greater adaptability
**Approx. Total Compute Time**	∼21,000 min ≈ 350 h (14.6 d) (1.5 min × 14,000 TO iterations)	∼1,838 min ≈ 30.6 h (1.28 d) (TO: 1,050 × 1.5 = 1,575 min; BO: 350 × 0.75 = 262.5 min)	≈10× faster

In terms of computational cost, each TO iteration, which involves both forward and adjoint simulations, takes approximately 1.5 minutes, while one BO evaluation, requiring only the forward simulation, takes about 45 seconds.

S2 Ablation Study on ViT Fine-Tuning Strategy

To empirically validate our choice of fine-tuning strategy for the Vision Transformer (ViT)-based VAE, as mentioned in Section 2.4 of the main text, we conducted a detailed ablation study. Our decision to unfreeze only the final two layers of the pre-trained ViT was initially guided by established best practices in transfer learning. The early layers of a ViT pre-trained on a large dataset like ImageNet capture general-purpose visual primitives

(e.g., edges, textures) that are highly transferable to other visual domains, including our metasurface patterns [S2]. Conversely, the later layers encode more abstract, dataset-specific features. By unfreezing only the final two transformer blocks, we enable the model to adapt to the specific visual grammar of our metasurface geometries while preserving the robust, low-level feature extractors while avoiding issues such as catastrophic forgetting [S2, S3].

To verify this, we compared the reconstruction quality across multiple fine-tuning strategies:

- Full Freeze: All 12 ViT transformer blocks are frozen.
- Partial Thaw: The final 1, 2, 4, or 6 transformer blocks are unfrozen for training.
- Full Fine-Tuning: All 12 ViT blocks are unfrozen.

The results of this study are summarized in Figure S2. We observed that a fully frozen encoder yields the highest validation reconstruction error (Mean Squared Error, MSE ≈ 0.0081). Allowing limited fine-tuning consistently improves performance, with the strategy of unfreezing the final two blocks achieving the lowest reconstruction error (MSE ≈ 0.0048). Unfreezing additional layers beyond this point did not yield further improvement and led to a slight degradation in performance, likely due to overfitting on our specialized dataset.

These results empirically confirm that our chosen strategy (last-two-block fine-tuning) provides the optimal trade-off between reconstruction fidelity and training stability, thus justifying its use in the main experiments. It must be noted that depending on the class of metaphotonic design problem and complexity of the selected structure and/or response, this number might slightly

change, but the general approach of using the last few layers is still the best practice.

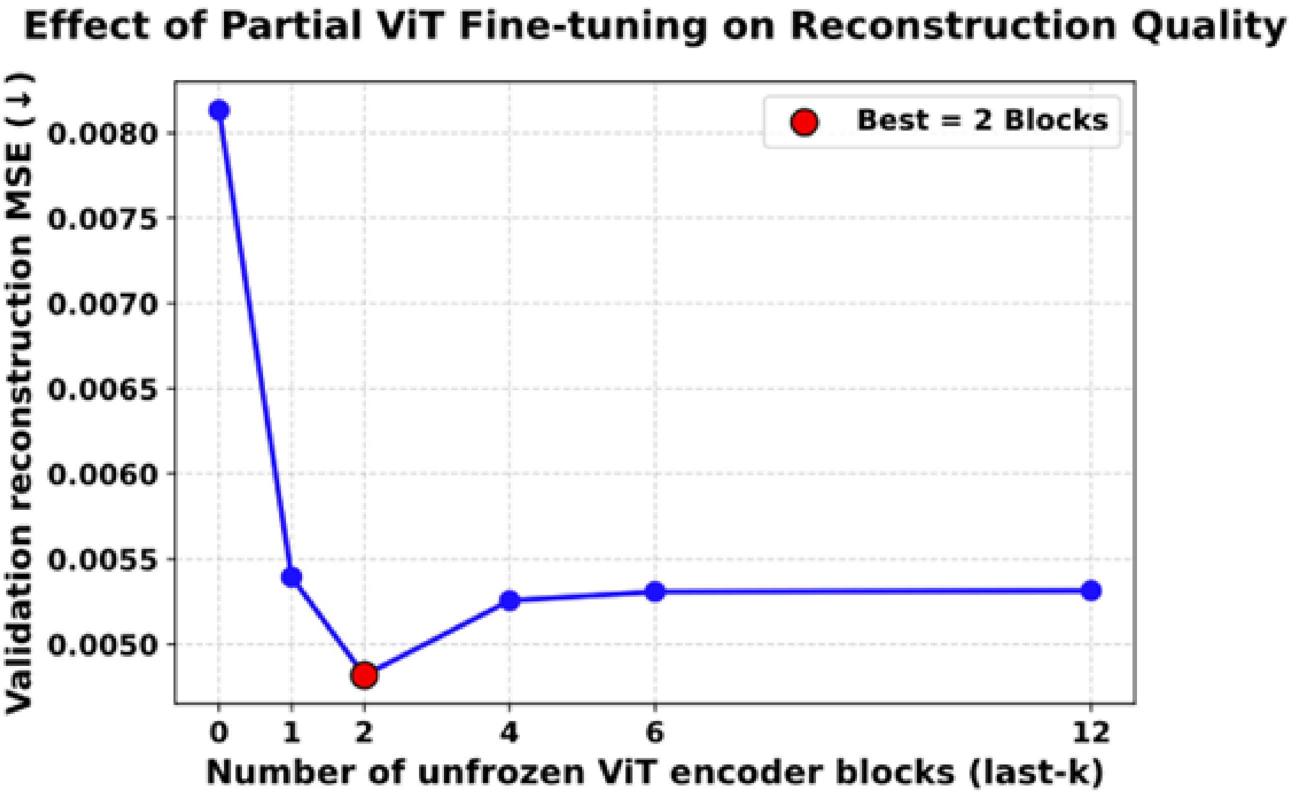

Figure S2: **Effect of partial ViT fine-tuning on reconstruction quality.** We compare the validation reconstruction MSE across different fine-tuning strategies, ranging from a fully frozen encoder (0 unfrozen blocks) to full fine-tuning (all 12 blocks). The optimal configuration is obtained by unfreezing only the last two ViT blocks, which yields the lowest reconstruction error while maintaining stability. This empirically validates our design choice reported in the main text.

S3 Hyperparameter Selection

This section provides a detailed explanation for the selection of key hyperparameters used throughout the HiLAB framework. These values were determined through a combination of established practices in literature, empirical tuning, and a coarse grid search aimed at optimizing performance for the specific problem of the achromatic beam deflector.

1. VAE Training and Post-Processing Parameters

These parameters govern the training of the generative VAE model and the final processing of the designs it produces.

- **KL Divergence Weight ($\alpha = 0.02$):** As defined in Equation (10) of the manuscript, this parameter regularizes the latent space. A small, non-zero weight is a common and critical practice in VAE training to encourage a smooth, continuous latent space while preventing posterior collapse, where the model ignores the latent variables [34, 38]. We found empirically that a value of 0.02 was sufficient to enforce a well-behaved latent space suitable for Bayesian optimization without overwhelming the primary goal of accurate reconstruction.

- **Binarization Penalty Weight ($\beta = 0.1$):** This term in the loss function encourages the VAE's direct output to be close to binary values (0 or 1). A coarse grid search revealed that a weight of 0.1 provided a good balance, effectively minimizing grayscale artifacts in the generated patterns—thus promoting manufacturability—without significantly degrading the reconstruction fidelity.

- **Post-Decoding Gaussian Smoothing ($\sigma = 2.0$ pixels):** As mentioned in Section 2.2 of the manuscript, a Gaussian filter is applied to the decoder's raw output. This is a standard technique to smooth out high-frequency noise and checkerboard artifacts often observed in generative models, ensuring the final patterns have clean, continuous features suitable for fabrication [33].

- **Final Binarization Threshold ($\tau = 0.6$):** This threshold converts the smoothed, grayscale image into a final binary pattern. While a value of 0.5 is a neutral midpoint, we empirically found that a slightly higher threshold of 0.6 produced patterns with sharper edges and fewer isolated island or hole

pixels, which are challenging for fabrication.

2. Topology Optimization (TO) Regularization Parameters

These parameters, defined in Equations (2) and (3), are crucial for guiding the initial TO runs toward smooth and manufacturable designs. The use of such filtering and projection techniques is a well-established practice in topology optimization for photonics [6, 29, 33].

- **Progressive Binarization (α_{min} = 1.0, α_{max} = 8.0, s = 10):** As shown in Figure S3(a), this schedule allows the design to exist in a grayscale state during early iterations, preventing premature convergence to a poor local optimum, while ensuring a strongly binary final pattern is achieved by the 35th iteration.
- **Cone Filter (r_{min}=1.0, r_{max}=2.5, k=15):** These values ensure that the spatial filter gradually increases its influence, starting with minimal smoothing to allow for broad exploration and ending with a larger radius to enforce a minimum feature size consistent with our fabrication constraints (Figure S3(b)).

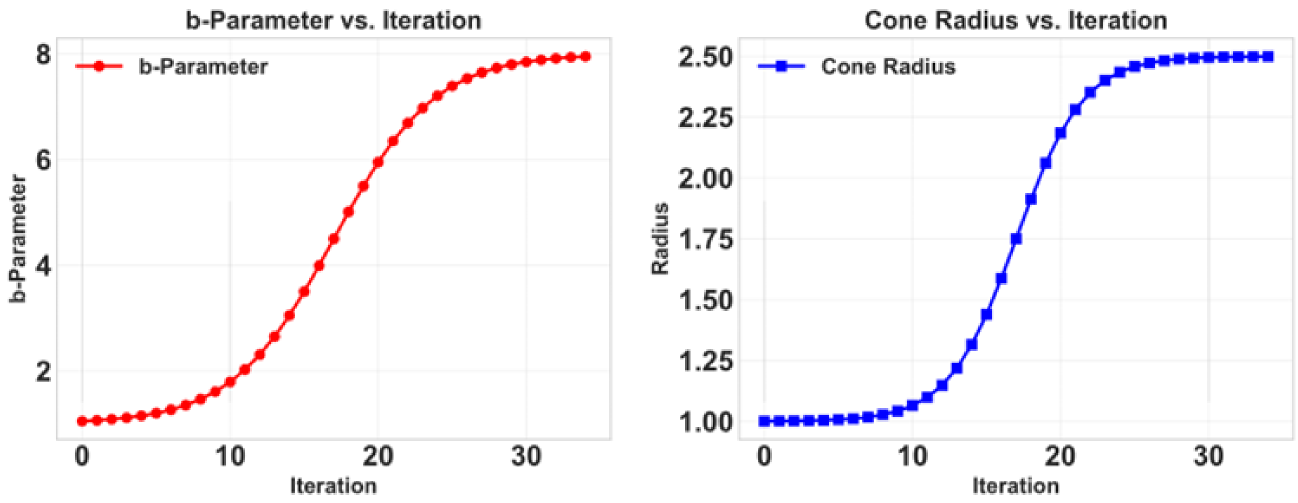

Figure S3: **TO Regularization Parameter Schedules.** Evolution of the progressive binarization parameter (α) (a) and the cone filter radius (b) as a function of the TO iteration number. Both parameters follow a logistic schedule, as defined in Equations (2) and (3) of the main text, respectively. The schedules are designed to allow for broad exploration in early iterations while gradually enforcing manufacturability constraints (smoothness and binarity) as the optimization progresses.

The specific numerical values for these TO regularization parameters were selected based on a combination of previous experience in designing freeform metasurfaces [33] and empirical tuning to ensure stable convergence for the present optimization task.

S4 Complex Refractive Index of TiO_2

The optical constants of TiO_2 were measured with a Woollam M-2000 variable-angle spectroscopic ellipsometer. A blanket TiO_2 thin film was deposited on a fused-silica wafer by electron-beam evaporation. Ellipsometry data were collected over the 400 nm to 1600 nm spectral range at multiple incident angles and fitted with a multi-oscillator dispersion model to extract both the real part, n, and the extinction coefficient, k, of the TiO_2 layer.

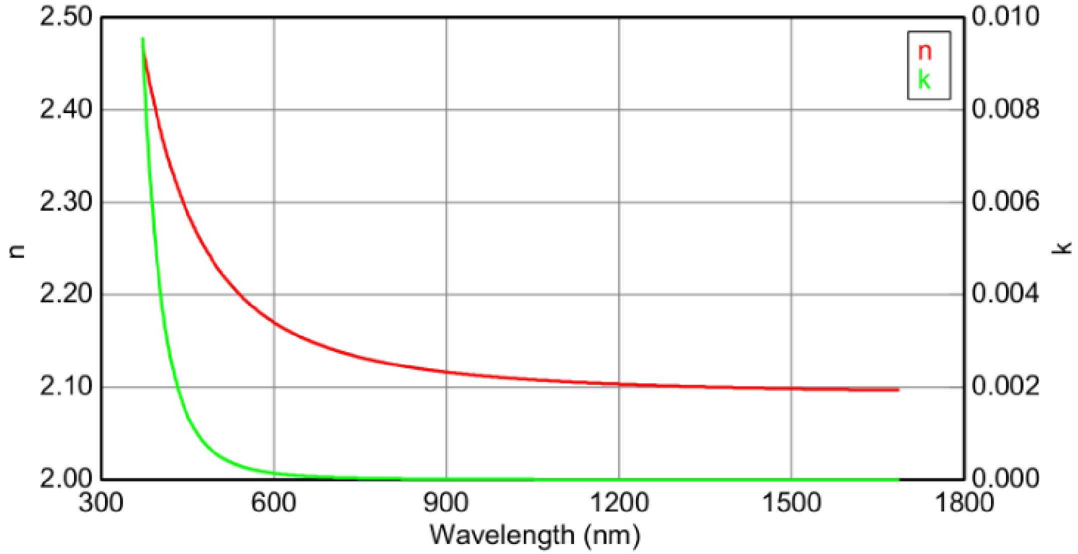

Figure S4: Measured complex refractive index of a 200-nm-thick TiO_2 thin film. The real part (n) and extinction coefficient (k) are plotted as functions of wavelength. The increase in k at shorter wavelengths indicates increased optical absorption in the blue region of the spectrum.

S5 Repeatability and Design-Space Navigation of HiLAB

Consistent with the convergence map shown in Figure 6 of the main paper, Supplementary Figure S5 (S5) documents a *second*

independent HiLAB run that begins from a fresh set of partial TO seeds. This additional experiment yields three major insights that reinforce the framework's robustness and practical utility. First, there is a repeatable discovery of high-FoM clusters: despite different random initial conditions, the Bayesian optimizer again concentrates sampling in a compact, high-performance pocket of the 11-dimensional space $\left[\mathbf{z}, \Lambda_y, t_1, t_2\right]$ after approximately 350 evaluations, mirroring the behavior reported in the main text. Second, we observe selective activation of latent coordinates: early iterations vary nearly all eight latent components, but by around iteration 280 only a subset remains active, indicating that the GP surrogate has *learned* which latent directions most strongly influence the FoM. This trait suggests a future knowledge-discovery avenue, whereby individual latent axes may be mapped to physical features of the metasurface. Finally, once a dense cluster of solutions is obtained, designers can make cluster, based design choices, trading performance for manufacturability by selecting, for instance, the member with the largest minimum feature size, without the need to rerun costly simulations.

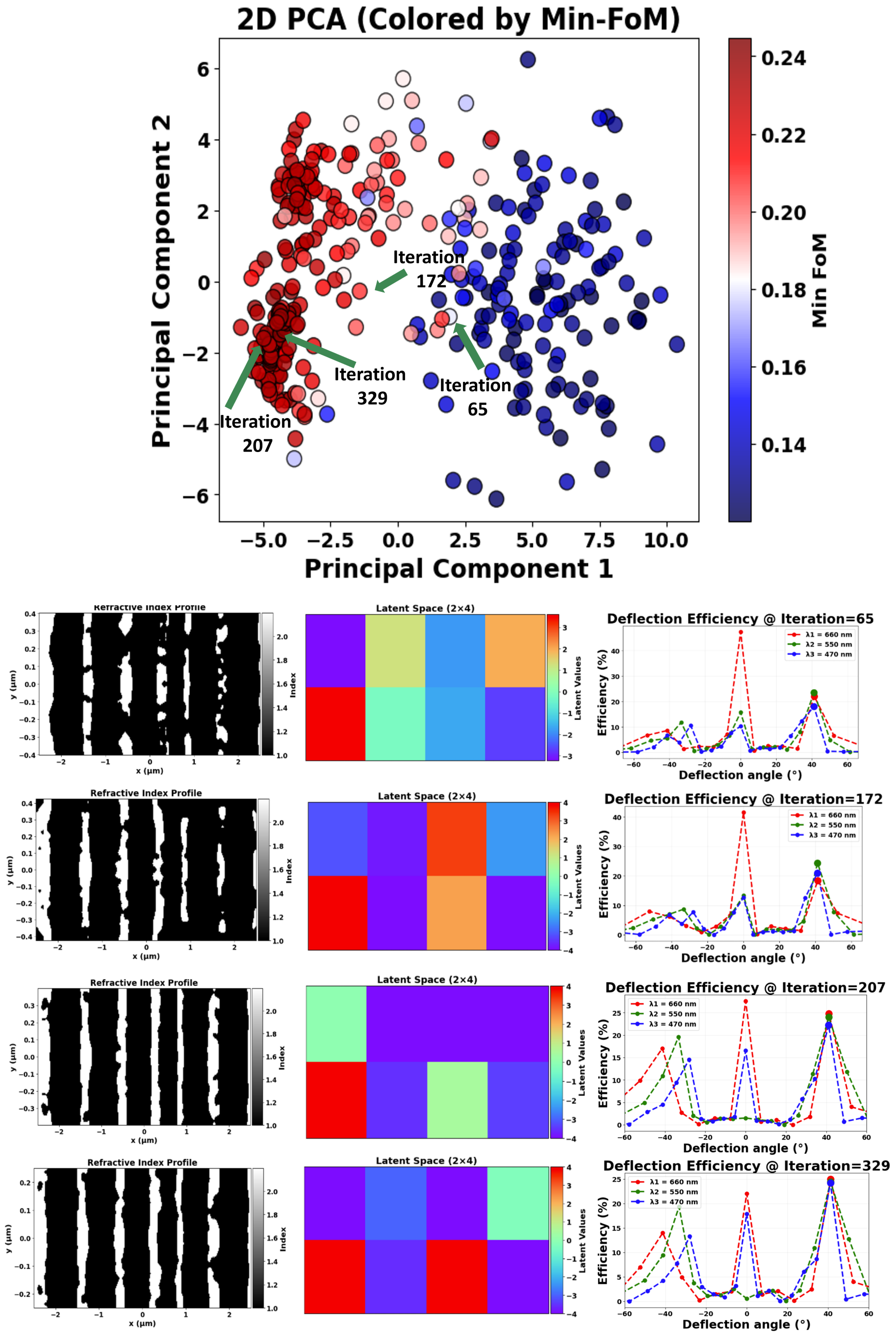

Figure S5: **HiLAB run demonstrating optimization repeatability and latent-space adaptation. Top:** Two-dimensional PCA projection of all evaluated designs, color-coded by the **minimum figure of merit (Min-FoM)** across the three target wavelengths. The trajectory shows clear convergence to a high-performing cluster, reproducing the behavior shown in Figure 6 of the main manuscript. **Bottom panels** (iterations 65, 172, 207, 329): *left*, binarized TiO_2/air index profile; *center*, corresponding 2×4 latent-space vector; *right*, angle-resolved diffraction efficiency spectrum, with the target deflection angle at 41.3°.

References

[S1] Dosovitskiy, A., Beyer, L., Kolesnikov, A., Weissenborn, D., Zhai, X., Unterthiner, T., ... & Houlsby, N. (2020). An image is worth 16x16 words: Transformers for image recognition at scale. *arXiv preprint arXiv:2010.11929*.
[S2] McCloskey, M., & Cohen, N. J. (1989). Catastrophic interference in connectionist networks: The sequential learning problem. In *Psychology of learning and motivation* (Vol. 24, pp. 109-165). Academic Press.
[S3] Kirkpatrick, J., Pascanu, R., Rabinowitz, N., Veness, J., Desjardins, G., Rusu, A. A., ... & Hadsell, R. (2017). Overcoming catastrophic forgetting in neural networks. *Proceedings of the national academy of sciences, 114*(13), 3521-3526.
